# Supplementary material for: Identification of Phlorotannins in the Brown Algae, Saccharina latissima and Ascophyllum nodosum by Ultra-High-Performance Liquid Chromatography Coupled to High-Resolution Tandem Mass Spectrometry
Source: Molecules. 2020 Dec 23;26(1):43. doi: 10.3390/molecules26010043 (PMC7795173; doi:10.3390/molecules26010043)
Supplement: Supplementary file 1 [file molecules-26-00043-s001.pdf]

Supplementary materials

A)

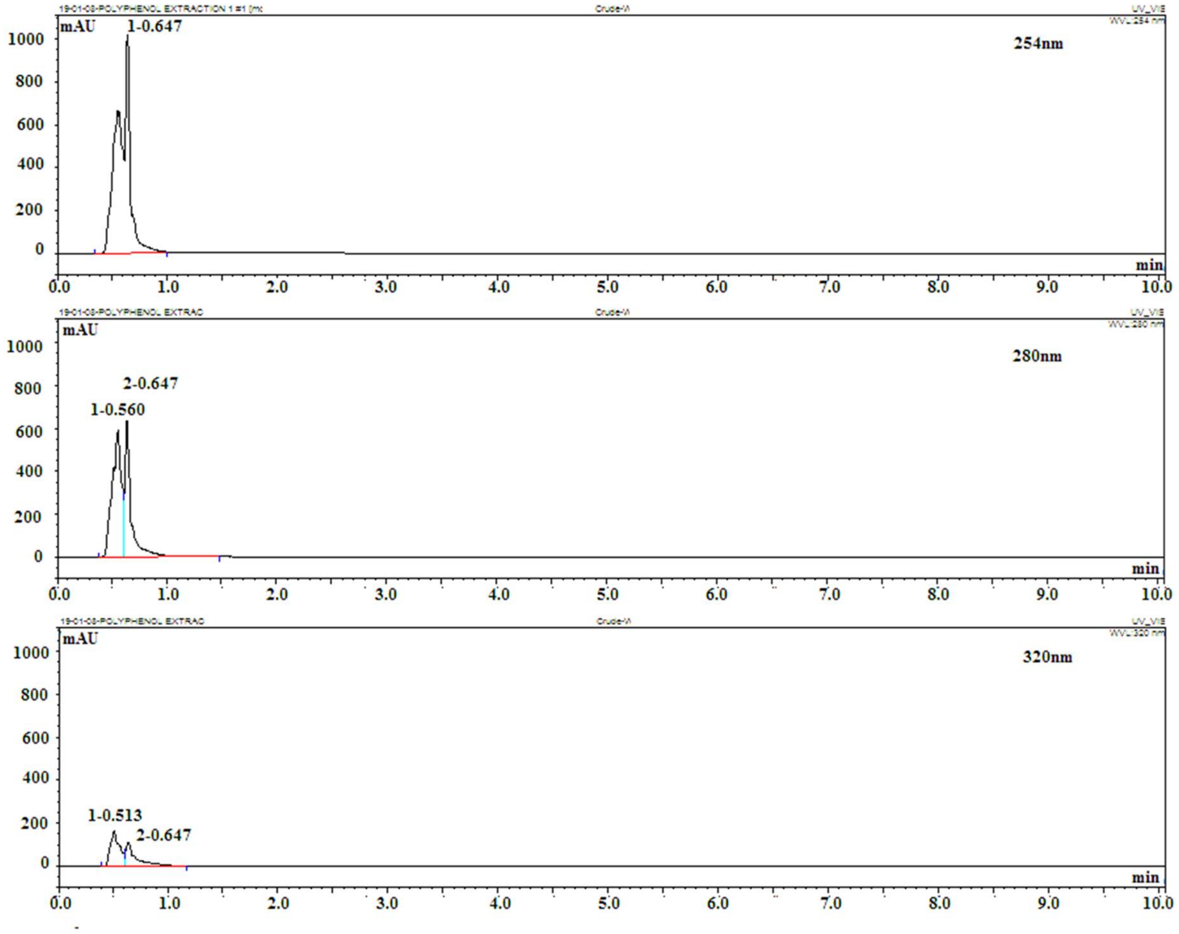

B)

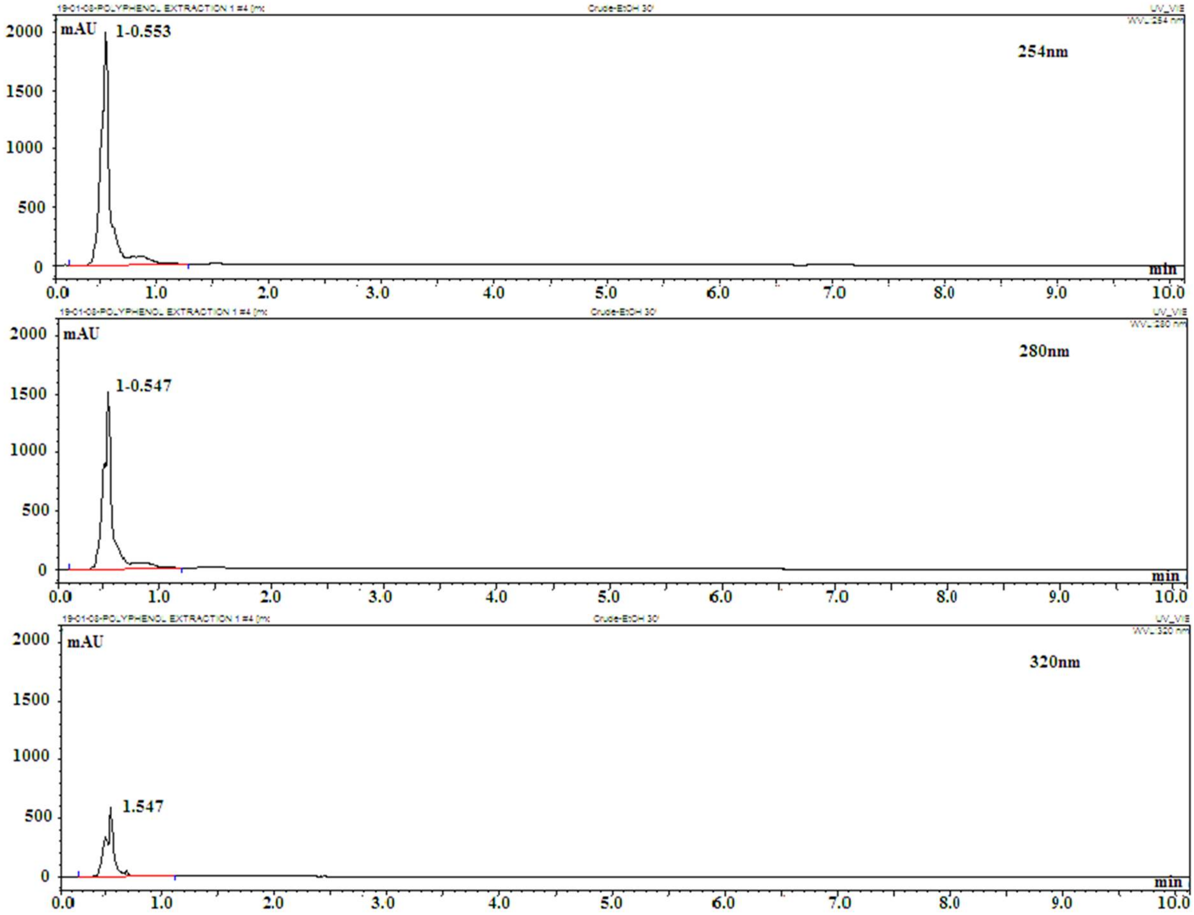

C)

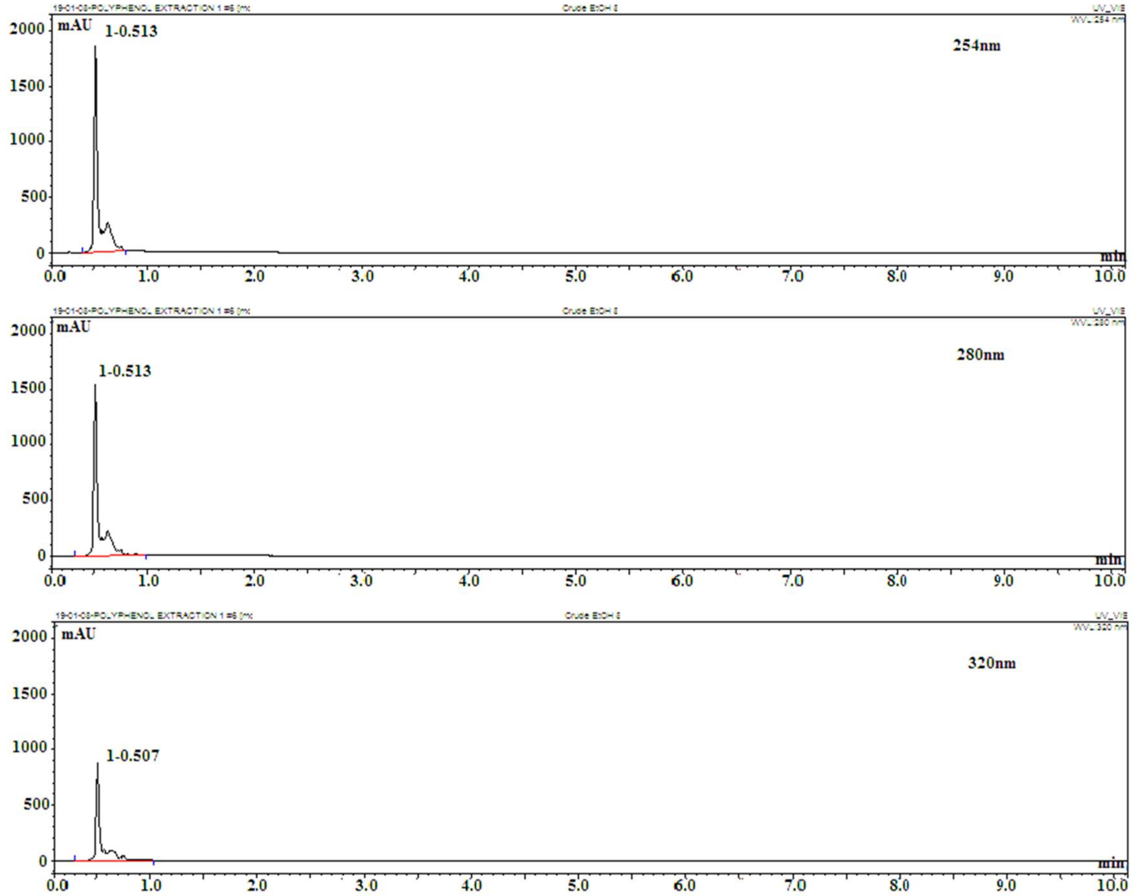

Figure S1: Chromatographic UHPLC profiles of crude extract of *S.latissima* extracted by (**A**) water, (**B**) aqueous ethanol (30% *v/v*), and (**C**) aqueous ethanol (80% *v/v*). The UHPLC profile was run at three different wavelengths.

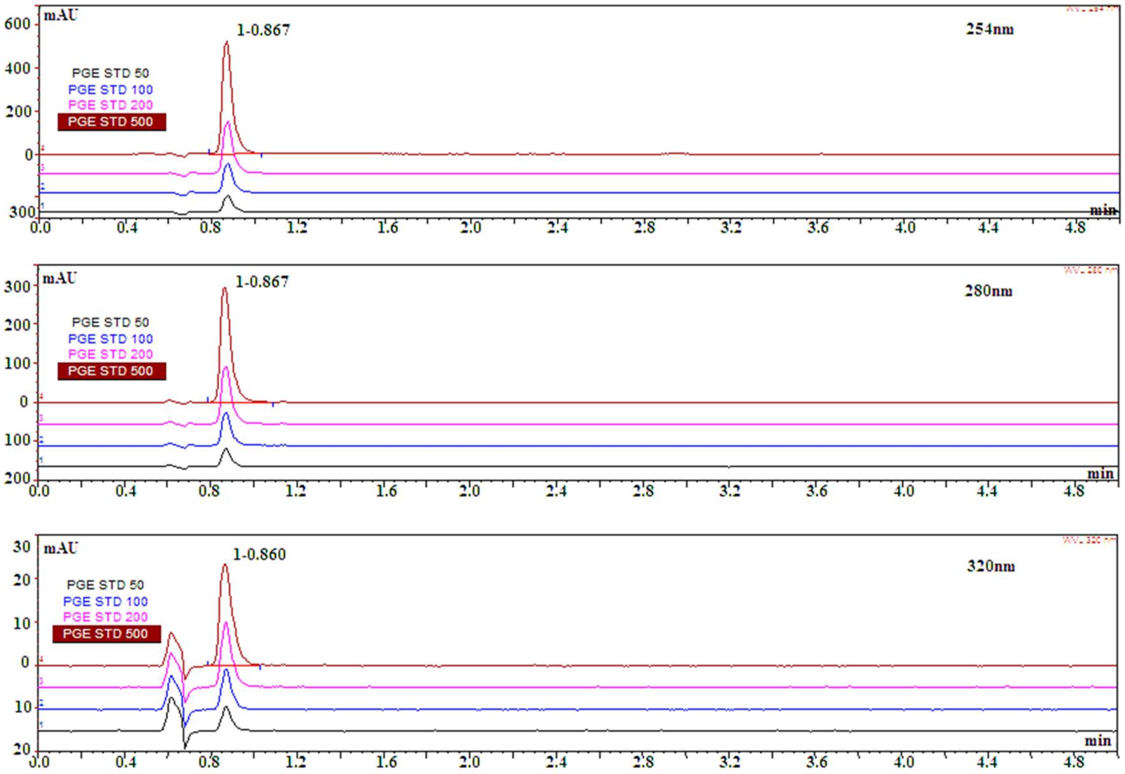

Figure S2: Chromatographic UHPLC profiles of phloroglucinol as standard at the concentrations of 50, 100, 200, and 500 mg/L. The UHPLC profile was run at three different wavelengths.

A)

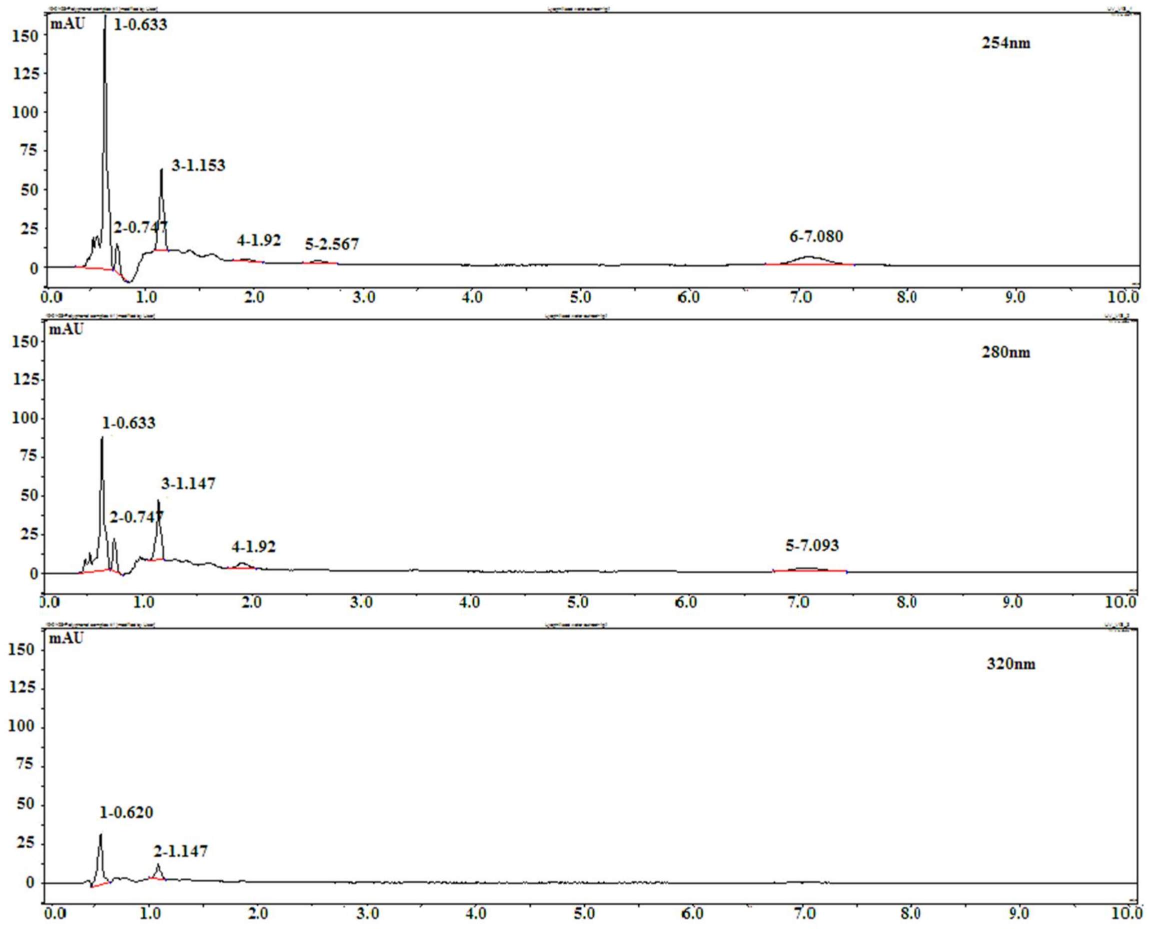
mAU
1-0.633
254nm
3-1.153
2-0.747
4-1.92
5-2.567
6-7.080
mAU
280nm
1-0.633
3-1.147
2-0.747
4-1.92
5-7.093
mAU
320nm
1-0.620
2-1.147
0.0
1.0
2.0
3.0
4.0
5.0
6.0
7.0
8.0
9.0
10.0

B)

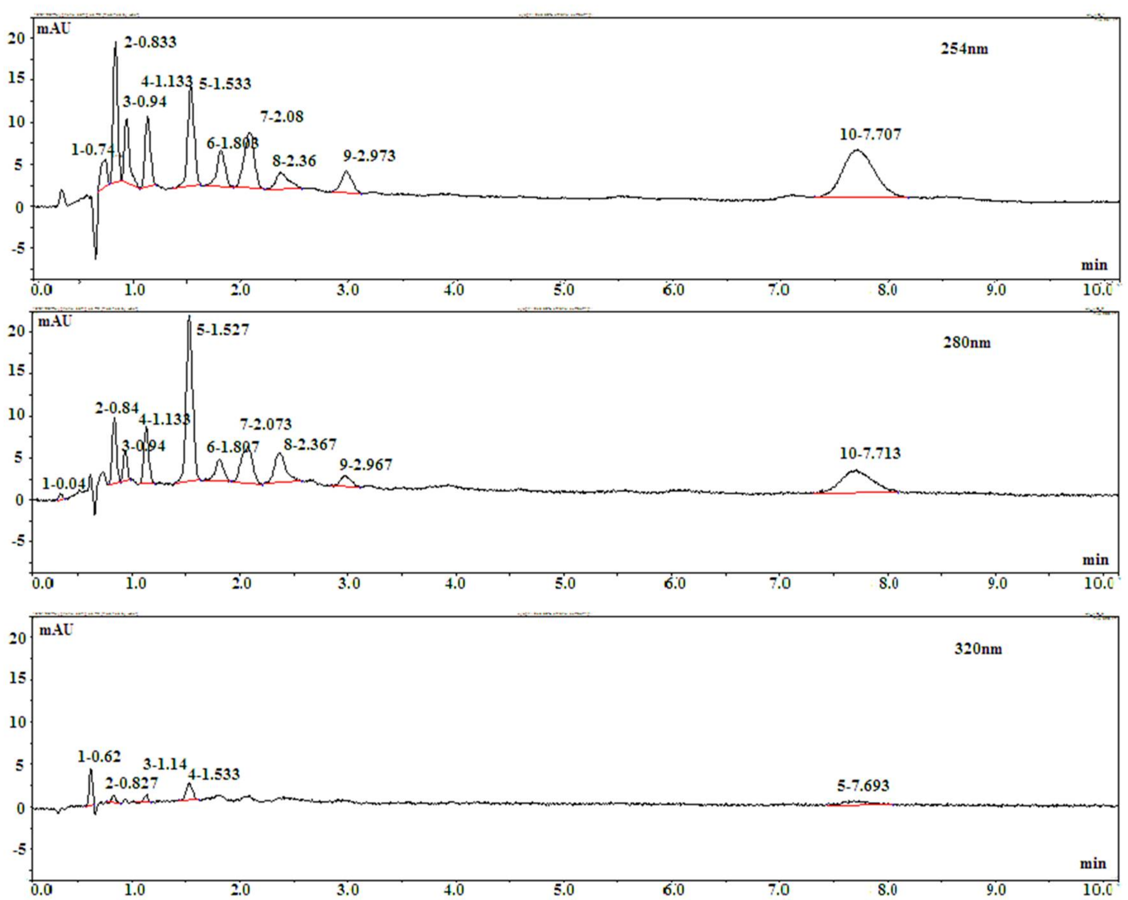
mAU
254nm
2-0.833
3-0.94
4-1.133
5-1.533
7-2.08
8-2.36
9-2.973
10-7.707
280nm
5-1.527
2-0.84
7-2.073
8-2.367
9-2.967
10-7.713
320nm
1-0.62
2-0.827
3-1.14
4-1.533
5-7.693
min
0.0
1.0
2.0
3.0
4.0
5.0
6.0
7.0
8.0
9.0
20
15
10
5
0
-5

C)

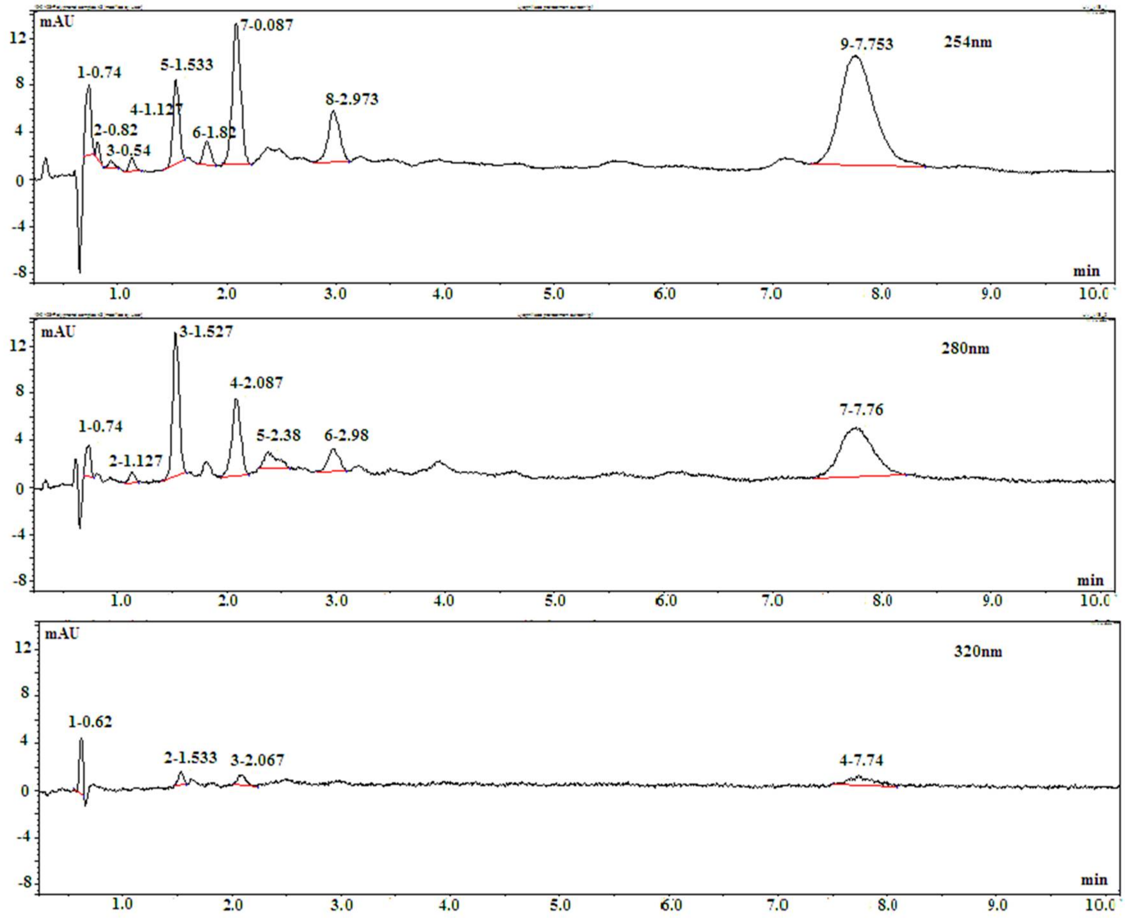

Figure S3: Chromatographic UHPLC profiles of lyophilized purified extracts (1 g/L) from *S. latissima*, extracted by (**A**) water, (**B**) aqueous ethanol (30% *v*/*v*), and (**C**) aqueous ethanol (80% *v*/*v*). The UHPLC profile was run at three different wavelengths.

.

A)

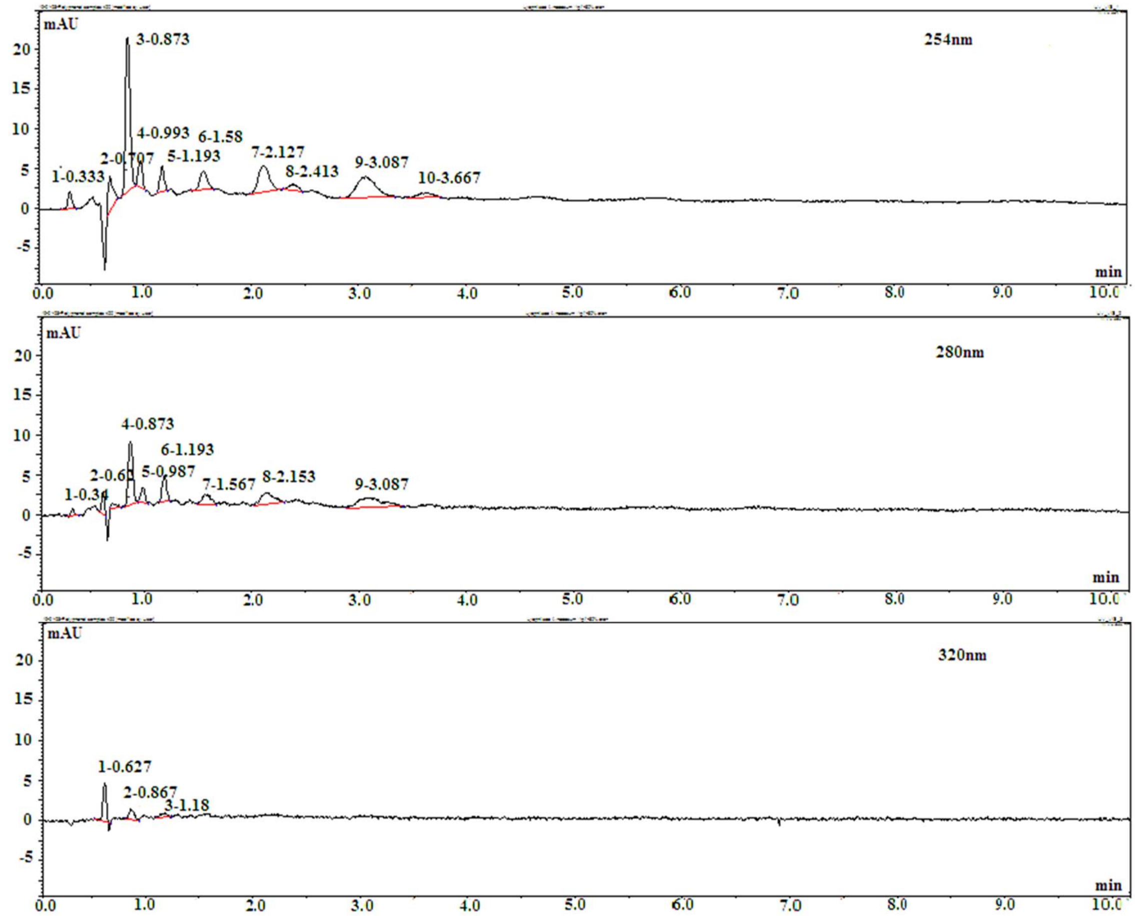
mAU
3-0.873
254nm
4-0.993
6-1.58
2-0.707
5-1.193
7-2.127
1-0.333
8-2.413
9-3.087
10-3.667
min
mAU
280nm
4-0.873
6-1.193
2-0.62
5-0.987
7-1.567
8-2.153
9-3.087
1-0.34
min
mAU
320nm
1-0.627
2-0.867
3-1.18
min

B)

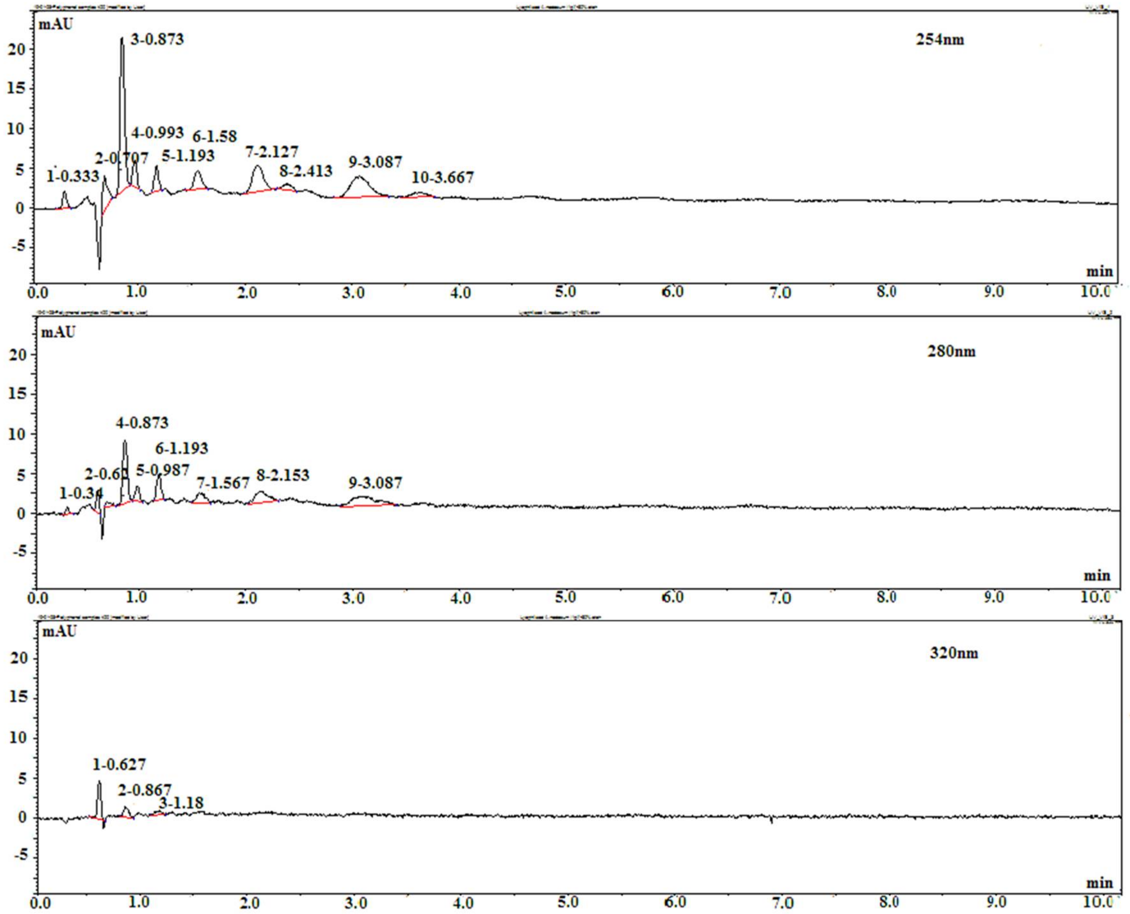

Figure S4: Chromatographic UHPLC profiles of lyophilized purified extracts (1 g/L) of *A. nodosum* extracted by (A) aqueous ethanol (30% *v/v*), and (B) aqueous ethanol (80% *v/v*). The UHPLC profile was run at three different wavelengths.

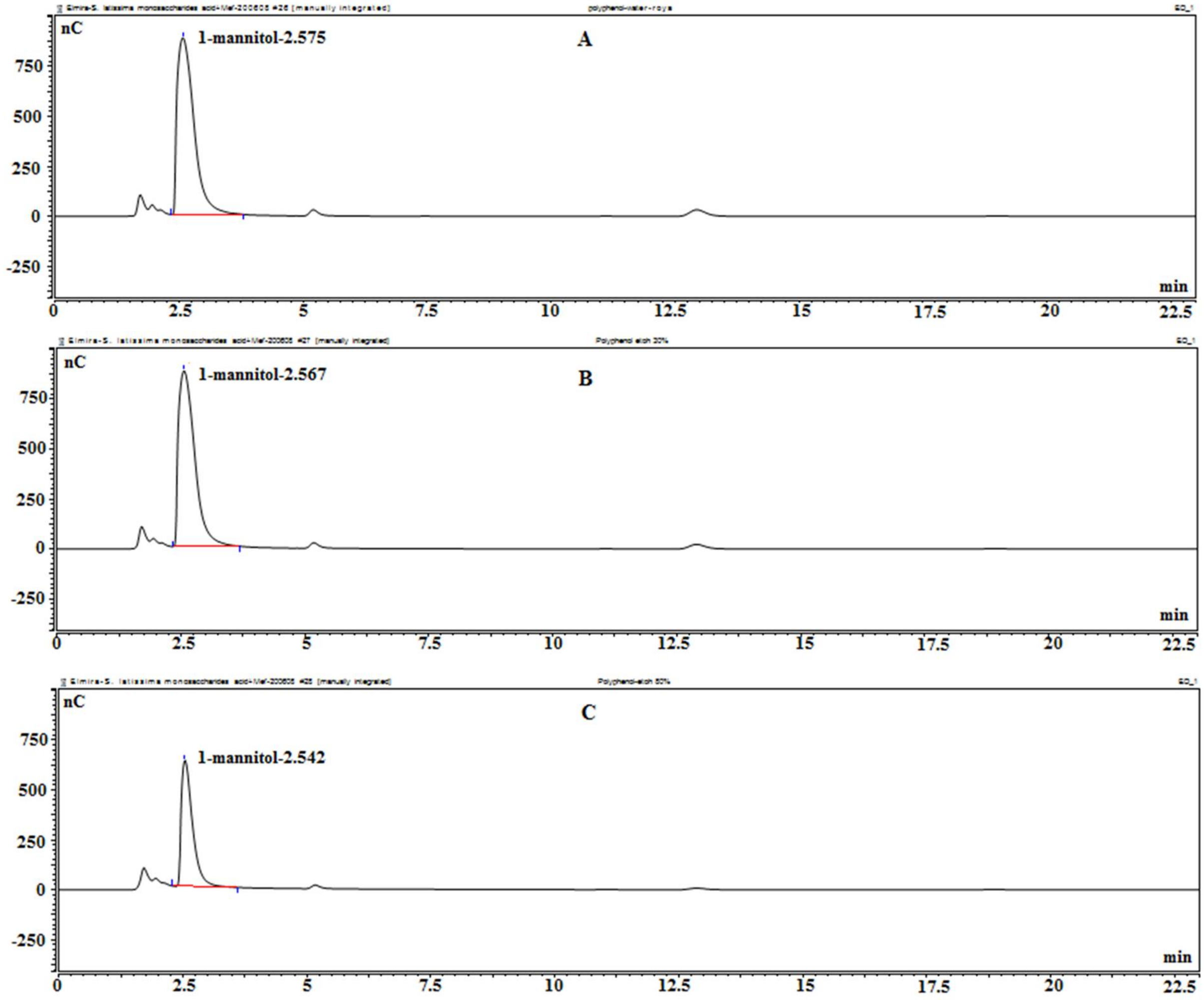

Figure S5: Chromatographic UHPLC profiles of sugars in extracts of *S. latissima* extracted by (**A**) water, (**B**) aqueous ethanol (30% *v*/*v*), and (**C**) aqueous ethanol (80% *v*/*v*).

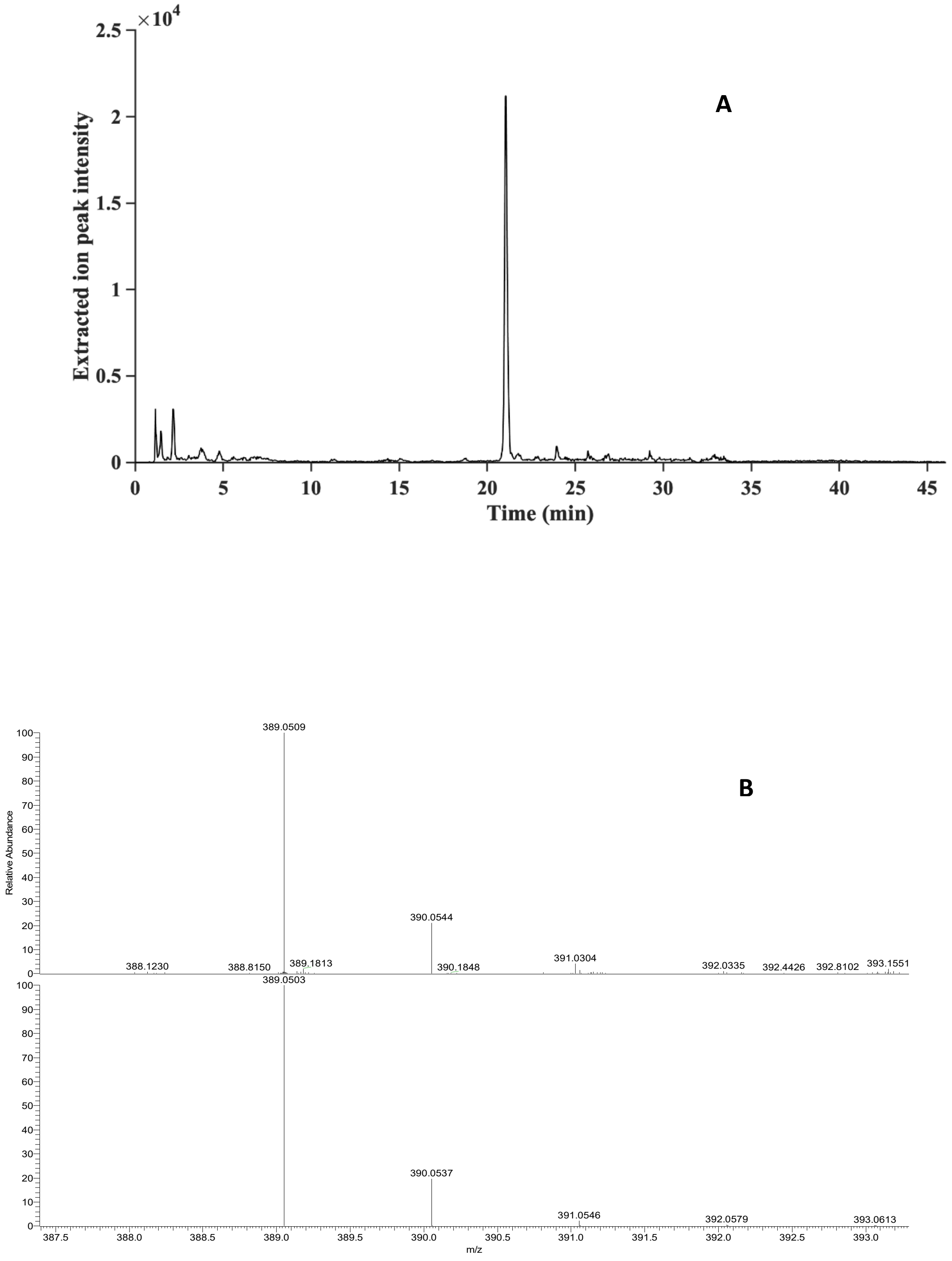

Figure S6: A. Extracted ion-chromatogram of *m/z* 389.0509 found in *A. nodosum*, **B.** Comparison of detected (top) and calculated (bottom) isotopes for *m/z* 389.0509 found in *A. nodosum*.

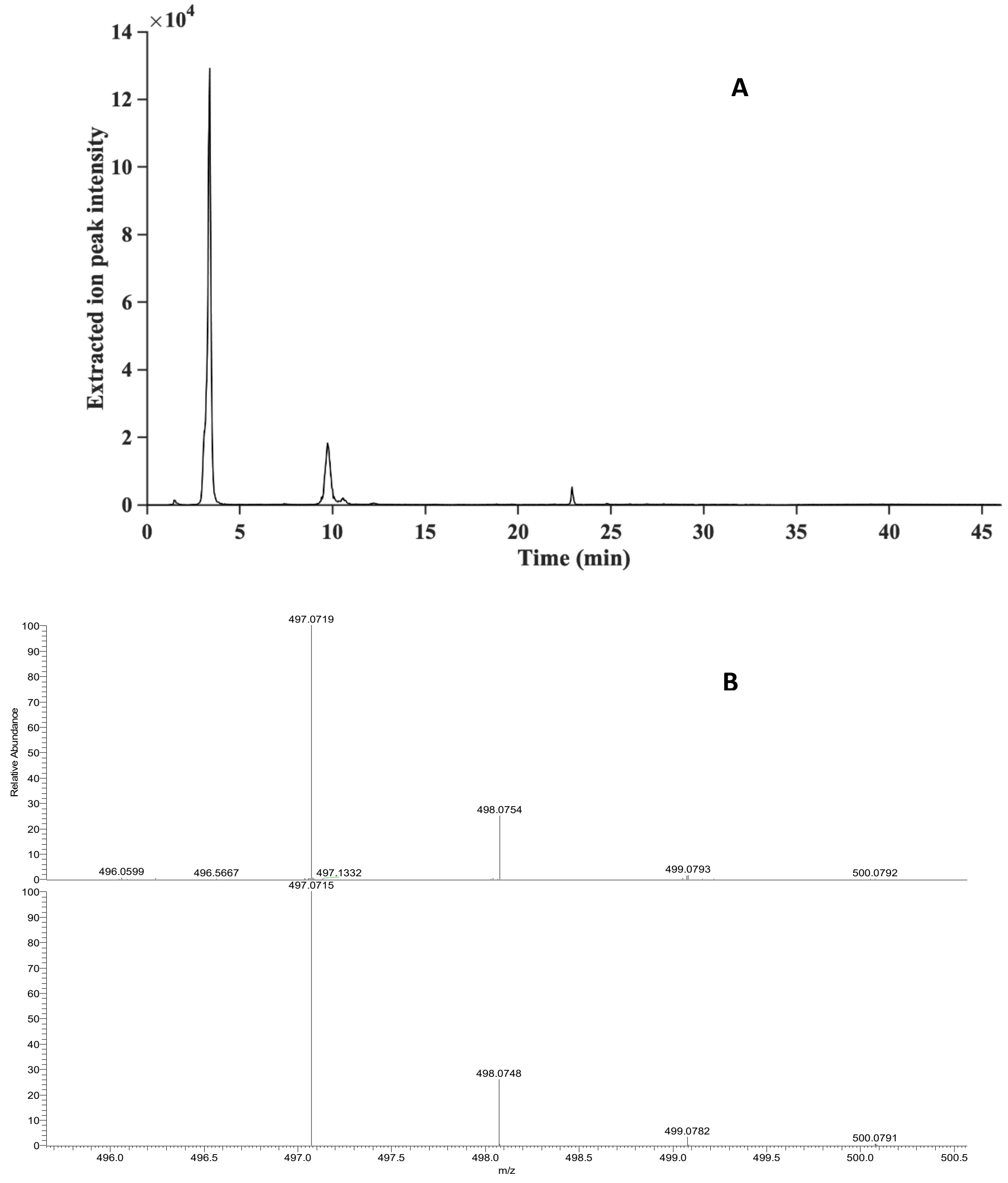

Figure S7: A. Extracted ion-chromatogram of *m/z* 497.0719 found in *A. nodosum*, **B.** Comparison of detected (top) and calculated (bottom) isotopes for *m/z* 497.0719 found in *A. nodosum*.

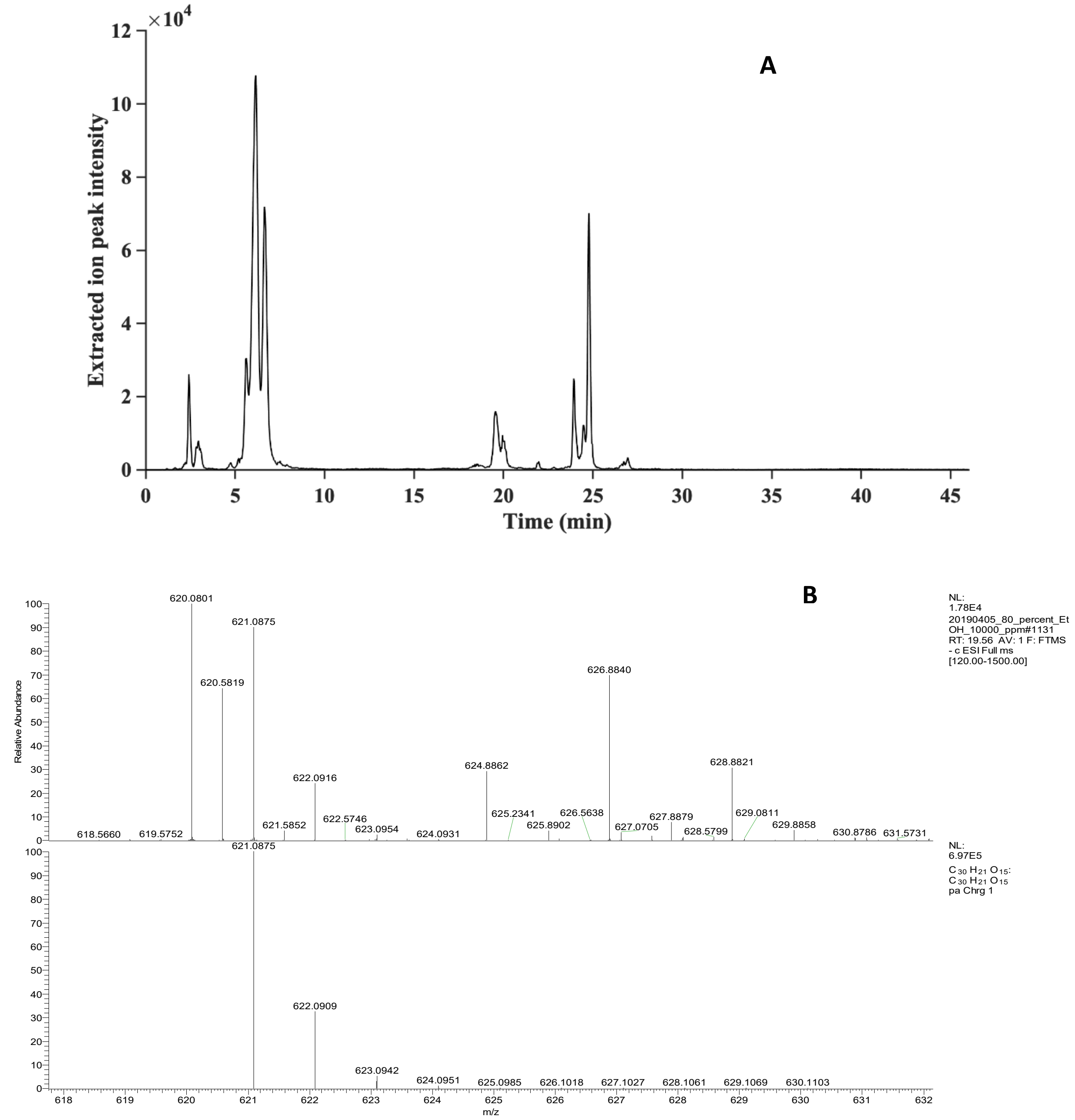

Figure S8: A. Extracted ion-chromatogram of *m/z* 621.0869 found in *A. nodosum*, **B.** Comparison of detected (top) and calculated (bottom) isotopes for *m/z* 621.0875 found in *A. nodosum*.

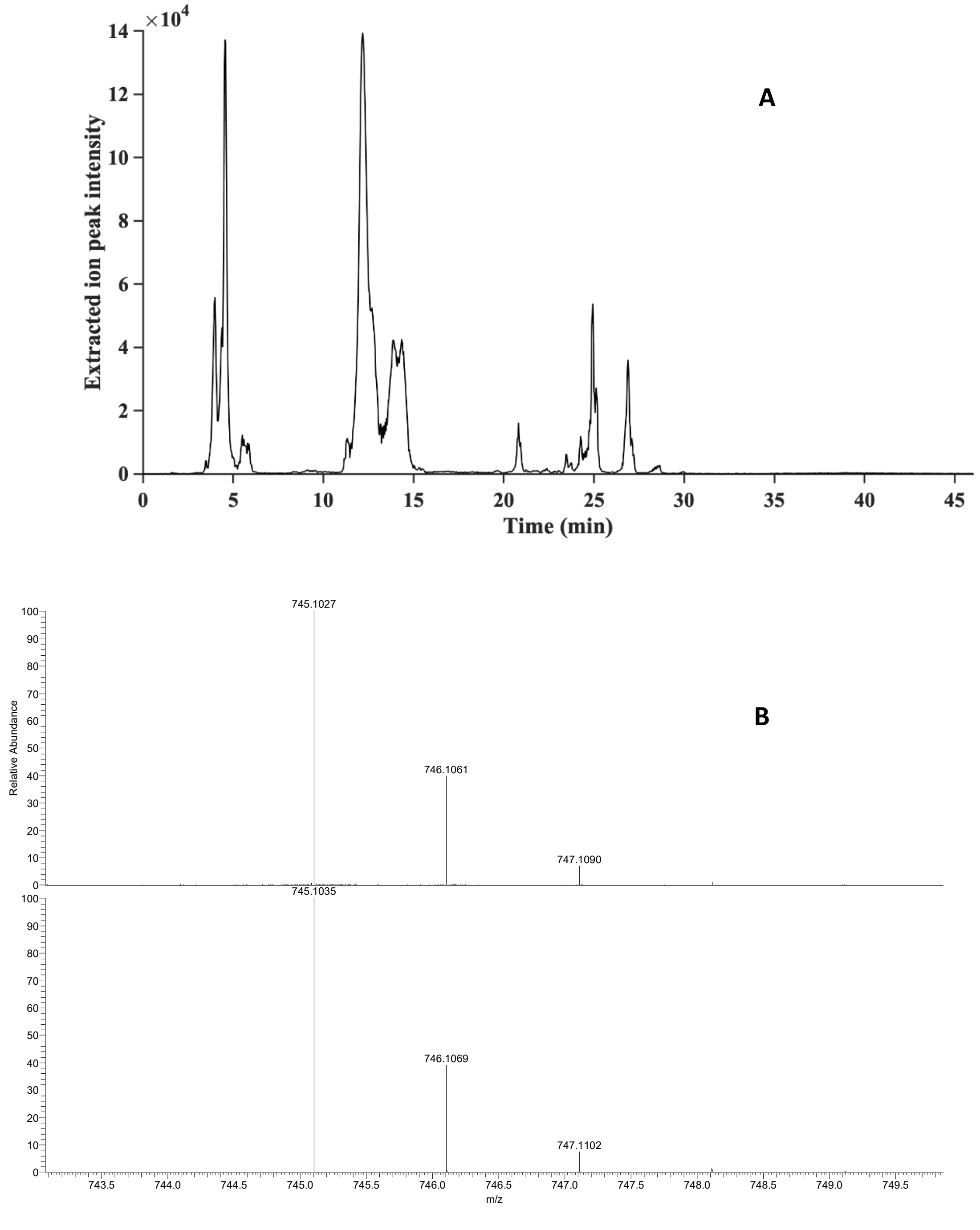

Figure S9: A. Extracted ion-chromatogram of *m/z* 745.1026 found in *A. nodosum*, **B.** Comparison of detected (top) and calculated (bottom) isotopes for *m/z* 745.1027 found in *A. nodosum*.

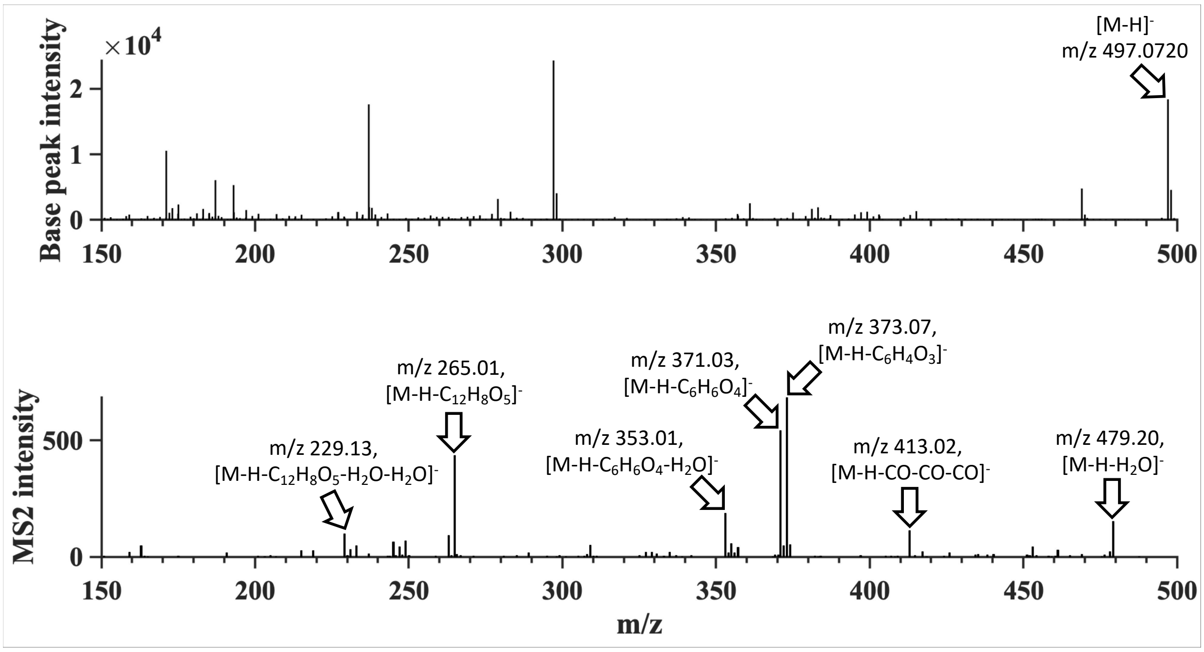

Figure S10: MS[1] and MS[2] spectra of *m/z* 497.0720 found in *A. nodosum*.

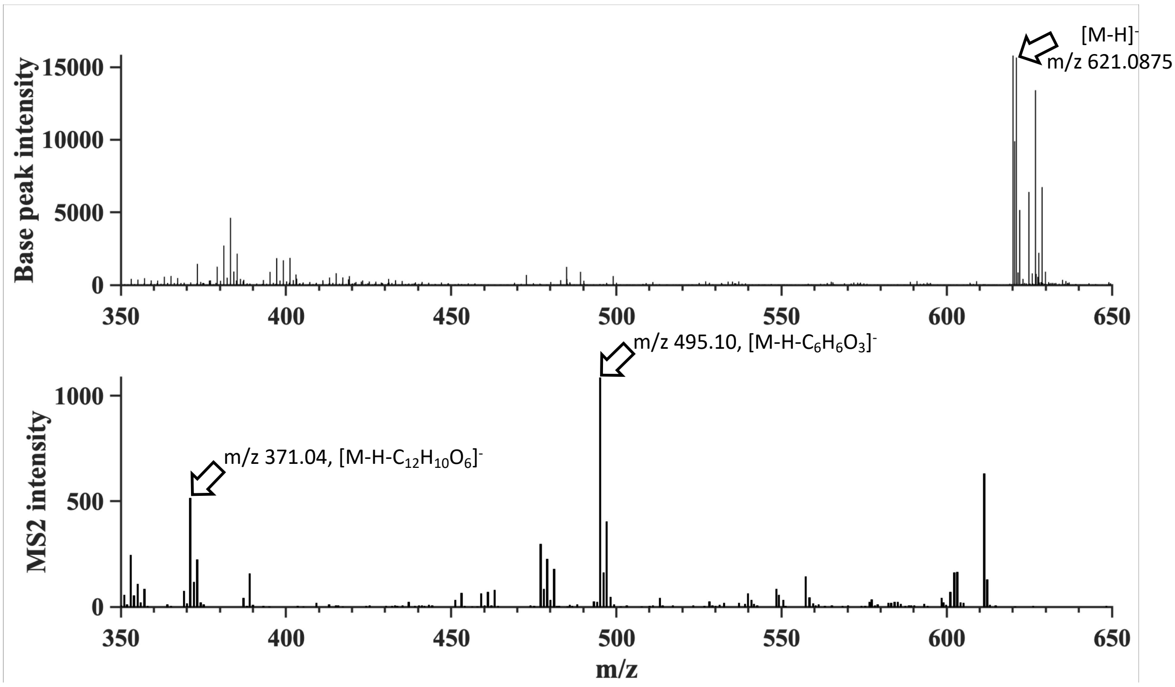

Figure S11: MS[1] and MS[2] spectra of *m/z* 621.0875 found in *A. nodosum*.

Table S1: MZmine 2 settings to create peak lists

1. Peak detection
   1.2 Mass detection function
      1.2.1 Settings:
         1.2.1.1 Polarity: negative
         1.2.1.2 Mass detector: exact mass
         1.2.1.3 Spectrum type: any
2. FTMS shoulder peaks filter
   2.2 Settings:
      2.2.1 Mass resolution: 100000
      2.2.2 Peak model function: Lorentzian extended
3. Chromatogram builder
   3.2 Settings:
      3.2.1 Min time span (min): 0.10
      3.2.2 Min height: 5.0E3
      3.2.3 *m/z* tolerance: 0.01 *m/z*
4. Chromatogram deconvolution
   4.2 Settings:
      4.2.1 Algorithm: local minimum search
         4.2.1.1 Chromatographic threshold: 80%
         4.2.1.2 Search minimum in retention time range (min): 0.20
         4.2.1.3 Minimum relative height: 1%
         4.2.1.4 Minimum absolute height: 5.0E3
         4.2.1.5 Min ratio of peak top/edge: 2
         4.2.1.6 Peak duration range (time): 0.00 – 10.00
      4.2.2 *m/z* center calculation: Median
5. Isotopic peak grouper
   5.2 Settings:
      5.2.1 *m/z* tolerance: 0.005 *m/z*
      5.2.2 Retention time tolerance: 0.2 min
      5.2.3 Maximum charge: 2
      5.2.4 Representative isotope: most intense
6. Chemical formula prediction
   6.2 Settings:
      6.2.1 Charge: 1
      6.2.2 Ionization type: [M-H]-
      6.2.3 *m/z* tolerance: 0.05 *m/z*
      6.2.4 Max best formulas per peak: 3
      6.2.5 Elements
         6.2.5.1 C (min: 0; max 100)

6.2.5.2 O (min: 0; max 100)

6.2.5.3 H (min: 0; max 100)

6.2.6 Element count heuristics

6.2.6.1 H/C ratio: on

6.2.6.2 NOPS/C ratios: off

6.2.6.3 Multiple element counts: on

6.2.7 RDBE restrictions

6.2.7.1 RDBE range: -1 to 40

6.2.7.2 RDBE must be an integer: yes

6.2.8 Isotopic pattern filter:

6.2.8.1 Isotope *m/z* tolerance: 0.005 *m/z*

6.2.8.2 Minimum absolute intensity: 1.0E3

Minimum score: 80.0%
